# Seroprevalence and Genetic Characterization of *Toxoplasma gondii* among Children with Neurodevelopmental Disorders in Egypt

**DOI:** 10.1155/2022/2343679

**Published:** 2022-05-27

**Authors:** Sara M. Elzeky, Nairmen Nabih, Aida A. Abdel-Magied, Dina S. Abdelmagid, Aya E. Handoussa, Marwa M. Hamouda

**Affiliations:** ^1^Department of Medical Parasitology, Faculty of Medicine, Mansoura University, Mansoura, Egypt; ^2^Department of Pediatrics, Faculty of Medicine, Mansoura University, Mansoura, Egypt

## Abstract

*Toxoplasma gondii* is a parasite with a special predilection for the central nervous system. Toxoplasmosis's contribution to the triggering of many neurodevelopmental disorders was established. This study aimed to detect the seroprevalence and genotypes of *T. gondii* strains in children with neurodevelopmental disorders. The study included 180 children with neurodevelopmental disorders and 180 children in the control group. Assessment of seropositivity of *Toxoplasma* IgM and IgG antibodies in patients and controls was carried out. Genetic characterization of *T. gondii* was obtained by nested polymerase chain reaction (PCR) and restriction fragment length polymorphism (RFLP) technique targeting dense granule gene (GRA6). Our results showed that the overall seroprevalence of *T. gondii* antibodies in the patient and controls was 35.6% and 11.7%, respectively. Nested PCR showed positivity in 11.1% of the patient group for *T. gondii* DNA. *T. gondii* seropositivity rate was significantly high in patients with hydrocephalus and also in patients with epilepsy. Positive nested PCR was significantly high in children with hydrocephalus only. Genotyping using nested PCR-RFLP showed genotype I (80%) followed by atypical strains (20%) with no association with any specific clinical presentation. In conclusion, among toxoplasmosis-positive children with neurodevelopmental disorders, analysis of *T. gondii* GRA6 locus revealed the predominance of type I genotype followed by atypical strains.

## 1. Introduction

Toxoplasmosis is a worldwide disease caused by the intracellular coccidian protozoan *Toxoplasma gondii* (*T. gondii*). This parasite can infect a wide range of warm-blooded animals in addition to humans, as intermediate hosts [1]. The infection occurs by ingestion of food contaminated with oocysts shed by the definitive host, felids, or undercooked meat contaminated with *Toxoplasma* tissue cysts. A congenital infection could occur through vertical transmission from the infected mother to the fetus [2].

Although toxoplasmosis could pass unnoticed in most adults, it can cause severe sequelae in immune-compromised patients; additionally, infection in pregnant women could cross the placental barrier and affect embryonic tissues [3]. Depending on the time of infection, vertical transmission could lead to miscarriage, microcephaly, hydrocephalus, and prematurity [4]. Postnatally acquired toxoplasmosis has a neurotropic localization mainly in the cerebral hemispheres, cerebellum, basal ganglia, and brain stem. This is followed by the formation of tissue cysts in neurons and glial cells leading to various psychomotor and neurological disabilities [5].

Diagnosis of toxoplasmosis relies on the detection of anti-*Toxoplasma* specific IgG and IgM antibodies using serological techniques such as enzyme-linked immunosorbent assay (ELISA) [6], in addition to molecular diagnosis such as polymerase chain reaction (PCR) [7].

*T. gondii* exhibits three main distinct typical clonal lineages known as genotypes I, II, and III [8]. More genetic variations of *T. gondii* were documented as atypical or recombinant genotypes. The severity of the three typical lineages in murine models differs from virulent type I to less virulent types II and III. In addition to the host immune status, the genotype of *T. gondii* could influence the course of the disease [9].

Genotype II was the most prevalent clonal type in Europe among immunocompromised individuals, and it was associated with both congenital and ocular toxoplasmosis [10]. Genotype I of *T. gondii* has been widely detected in severe congenital toxoplasmosis and in immunocompetent cases [11]. While typical clonal types were prevalent in Europe and North America, atypical diversities of *T. gondii* were isolated from South America [12].

Outlining the biological populations of *T. gondii* is vital in tracing the infection source for epidemiological surveys. Furthermore, defining a specific isolate in human toxoplasmosis is vital for vaccine development and consequently disease control [13].

Pathogenicity analysis has shown that different disease outcomes in different *T. gondii* strains might be due to polymorphisms in parasite-derived effector proteins originating from apical secretory organelles, such as microneme proteins (MICs), rhoptry proteins (ROPs), surface antigen 2 gene (SAG2), and dense granule proteins (GRAs) [14]. A single-copy gene with a high degree of sequence polymorphism encodes a GRA protein, called GRA6 [15]. Analysis of this gene could be used for differentiation between the three *T. gondii* genotypes, as well as some of the atypical strains [16].

This study aimed to detect the seropositivity rate of *T. gondii* infection in children with neurodevelopmental disorders and to demonstrate the genotypes of *T. gondii* isolated from those children. Nested PCR-RFLP using the GRA6 gene was used for polymorphism identification.

## 2. Materials and Methods

### 2.1. Study Participants

This was a case-control study, including 180 children aged from birth up to 11 years, with different neurodevelopmental disorders attending the Neurology Department outpatient clinics, Mansoura University Children Hospital, Mansoura, Egypt (patient group). Neurodevelopmental disorders included hydrocephalus, microcephaly, cerebral palsy, epilepsy, and mental retardation, and the diagnosis of the cases was carried out in the Neurology Department. In addition, 180 children with no history of obvious CNS disorders were randomly chosen from children attending Mansoura University Children's Hospital laboratory for health screening or as visitors to the patients (control group). History of head trauma, brain surgery, malignancy, and family history of consanguinity, congenital anomalies, or neuropsychiatric diseases were excluded. A preplanned questionnaire with full history was completed for each child for demographic data, associated risk factors, and neurodevelopmental history.

### 2.2. Ethical Consideration

The study protocol was approved by the Institutional Review Board (IRB) of the Faculty of Medicine, Mansoura University, Mansoura, Egypt (Approval code: MD.19.01.122.R1). Informed written consent was obtained from each participant´s parent/guardian in the study, involving a voluntary decision about whether to participate or not with respecting the confidentiality of their data. The study was conducted in accordance with the guidelines of the Helsinki Declaration as revised in 2013.

### 2.3. Blood Sampling

Blood samples were collected from patient and control groups under aseptic conditions. Each sample was divided into two tubes (3 ml each): one was for serum extraction after centrifugation at 3000 rpm for 10 min and the other one contained EDTA for extracting the DNA. Both tubes were kept a −20°C until laboratory examination.

### 2.4. Detection of Anti-*Toxoplasma* IgG and IgM Antibodies

Both anti-*Toxoplasma* IgG and IgM antibodies were evaluated for all serum samples using a commercially ELISA kit (Biocheck Inc., CA, USA), according to the manufacturer's instructions. The sensitivity and specificity of the kit were 98.3% and 99.2%, respectively. The absorbance of each sample was measured using a microwell reader with a 450 nm filter (Chromate Reader, USA). *Toxoplasma* index less than 0.90 was considered negative, and that between 0.91 and 0.99 was equivocal and must be retested; however, a *Toxoplasma* index of 1.00 or greater was considered positive for anti-*Toxoplasma* antibody. For quantitative estimation of anti-*Toxoplasma* IgG levels of positive specimens as IU/ml, OD of cut-off and calibrators were plotted on *Y*-axis in a graph versus their corresponding anti-*Toxoplasma* IgG concentrations of 0, 32 (cut-off), 100, and 300 IU/ml on *X*-axis. The *Toxoplasma* IgG levels in patient and control sera were interpreted from the standard curve using their individual OD values.

### 2.5. DNA Extraction and PCR Amplification GRA6 Gene

The genomic DNA was extracted from whole blood using Gene JET genomic DNA Purification Kit (Thermo Scientific, USA) according to the manufacturer's instructions. A nested PCR was used for amplification of the coding region of the GRA6 gene [12], using the thermal cycler (TECHEN TC-312, UK). Nested PCR protocol was carried out with external and internal primers (Biosearch Technologies, USA) in two subsequent amplifications. The first amplification was done using the forward external primer (5-GGC AAA CAA AAC GAA GTG-3) and the reverse external primer (5-CGA CTA CAA GAC ATA GAG TG-3). In the second amplification, the forward internal primers (5-GTA GCG TGC TTG TTG GCG AC-3) and reverse internal primers (5-TAC AAG ACA TAG AGT GCC CC-3) were used [17]. The first PCR amplification was carried out in a 25 *μ*l reaction volume containing 5 *μ*l of DNA extract, 12.5 *μ*l of Red Taq® Ready Mix (Sigma Aldrich, USA), 0.25 *μ*l of each forward and reverse external primer, and 7 *μ*l of nuclease-free water. The amplification was performed in the following steps: the first cycle of denaturation at 94°C for 5 min, 35 cycles of denaturation at 94°C for 30 s, annealing at 54°C for 1 min, and extension at 72°C for 90 s, and a final extension step at 72°C for 7 min. The resulting amplification products were diluted in an equal amount of nuclease-free water. The second-round PCR was conducted in a reaction mixture containing 25 *μ*l of Master Mix, 10 *μ*l of the diluted PCR product, 0.5 *μ*l of each forward and reverse internal primer, and 14 *μ*l of nuclease-free water. The steps of PCR amplification included initial denaturation at 94°C for 5 min, 35 cycles of denaturation at 94°C for 30 s, annealing at 60°C for 60 min, extension at 72°C for 90 s, and finally a 7 min extension step at 72°C [18]. A 10 *μ*l of nested PCR DNA products was subjected to electrophoresis with a 100 base pair DNA ladder (Thermo Fisher Scientific, USA) in 2% agarose gel, stained with ethidium bromide, and visualized under UV illumination.

### 2.6. Genotyping by Restriction Fragment Length Polymorphism (RFLP)

All positive samples by nested PCR using the GRA6 coding region, as the target gene, were processed by the MseI restriction enzyme, Fast Digest® Saq AI (Thermo Scientific, USA). According to the manufacturer's instructions, digestion was performed at 37°C for 15 min in a 30 *μ*l volume per reaction with 1 *μ*l of restriction enzyme, 2 *μ*l fast digest buffer, 10 *μ*l of the PCR product, and 17 *μ*l of nuclease-free water. For visualization of the digestion products, it was loaded directly on 2% agarose gel electrophoresis stained with ethidium bromide. *T. gondii* strain genotype was determined by comparing the restriction pattern to that described by Fazaeli et al. [16] as differentiation of parasite strains was done by their cutting products (168 and 544 bp, 75 and 623 bp, and 97 and 544 bp fragments in type I, type II, and type III, respectively).

### 2.7. Statistical Analysis and Data Interpretation

SPSS software version for Windows version 22.0, Armonk, USA, was used for statistical analysis of the results. Qualitative data were described using numbers and percentages. Quantitative data were analyzed using means and standard deviations. Chi-square and Fisher's exact tests were used to compare categorical variables. The Mann–Whitney *U* test was used to compare two independent groups. Logistic regression analysis was performed for the association between risk factors and toxoplasmosis seropositivity. The significance of the obtained results was judged at the 0.05 level.

## 3. Results

**Figure 1:**
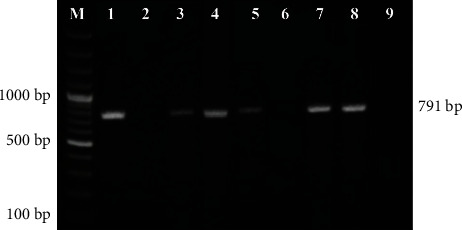
Gel electrophoresis of nested PCR products for amplification of Toxoplasma gondii GRA6 gene. M: 100 bp molecular weight marker. Lane 1: positive control (Toxoplasma RH strain); lane 2: negative control (nuclease-free water); lanes 3, 4, 5, 7, and 8: positive nested PCR samples showed amplification at GRA6 gene-specific bands (791 bp). Lanes 6 and 9: negative samples.

As demonstrated in Table 1, the overall seroprevalence of *T. gondii* antibodies in children with neurodevelopmental disorders (patient group) and controls was 35.6% and 11.7%, respectively (*P* < 0.001). The seropositivity of the anti-*Toxoplasma* IgM antibodies was detected in 10% of the patient group and in 1.1% of the control group, with a significant difference between both groups (*P*=0.04). The seropositivity of the anti-*Toxoplasma* IgG antibodies was significantly higher in the patient group (23.3%) compared to the controls (10%), (*P* < 0.001). Furthermore, considering a titer of 32 IU/ml as a cut-off point, the median of anti-*Toxoplasma* IgG antibodies concentration titer was significantly higher in the patient group (309.83 IU/ml) compared to the median detected in the controls (61.13 IU/ml), (*P* < 0.001). Combined IgM/IgG antibodies were detected in 2.2% of the patient group and in 0.5% of the controls. Nested PCR amplification of the GRA6 gene of *T. gondii* showed an 11.1% positivity rate in the patient group (Figure 1) and 0.5% in controls (*P*=0.003).

The relation between toxoplasmosis positivity rate and different risk factors among patient and control groups was illustrated in Table 2. The neonate age group was less than 28 days old, the infant age group was between 28 days and one year old, and the child age group was from one up to 11 years old. In children with neurodevelopmental disorders, the seropositivity of *T. gondii* was higher in the neonatal age group (48.6%) than in the child (36.7%) and infantile (21.4%) age groups, although a nonstatistically significant difference was detected. There was a significantly high PCR positivity rate in the neonatal age group (*P*=0.009).

The risk for toxoplasmosis was significantly higher in patients (Odd Ratio (OR), 95% confidence interval (CI): 4.3, 2.12–9.7; *P* < 0.001) and controls (OR, 95% CI: 2.15, 1.06–4.39; *P* < 0.001) who lived in rural areas than those who lived in urban areas. In the patient group, high anti-*Toxoplasma* antibodies positivity rate was detected in association with a history of contact with soil (OR, 95% CI: 6.59, 3.2–13.7; *P* < 0.001) and contact with cats (OR, 95% CI: 1.48, 0.57–3.84; *P*=0.008). Blood transfusion was recorded in 5 cases (all were child age group), from which only one case was positive for anti-*Toxoplasma* IgG.

Table 3 showed the relation between *T. gondii* positivity rate and different neurodevelopmental disorders. Anti-*Toxoplasma* antibodies and PCR positivity rates were significantly high in children with hydrocephalus (69.2% and 26.9%, respectively) (*P* < 0.05). Moreover, *T. gondii* seropositivity was significantly high in epilepsy patients (60.8%) (*P* < 0.001).

**Figure 2:**
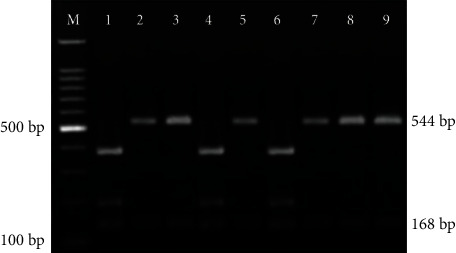
Agarose gel electrophoresis of MseI restriction enszyme digestion of Toxoplasma gondii GRA6 gene nested PCR amplification products. M: 100 bp molecular weight marker. Lanes 2, 3, 5, 7, 8, and 9: samples coinciding with type I (cutting bands at 168, 544 bp). Lanes 1, 4, and 6: atypical digestion pattern.

Characterization of *T. gondii* genotypes in nested PCR-positive samples was performed using RFLP. *T. gondii* genotypes were determined by comparing the digestion pattern after digestion of amplification products with the MseI restriction enzyme (Figure 2). Out of twenty positive PCR samples from children with neurodevelopmental disorders, 80% (16/20) had a strain pattern coinciding with *T. gondii* genotype I, and 20% (4/20) displayed an atypical RFLP pattern (Table 4). On the other hand, the positive PCR sample from the control group had shown no digestion pattern.

## 4. Discussion

In the present study, the seropositivity rates of anti-*Toxoplasma* IgM, IgG, and combined IgM/IgG in children with neurodevelopmental disorders were 10%, 23.3%, and 2.2%, respectively. In our study, the seropositivity rates of anti-*Toxoplasma* antibodies were significantly higher in children with neurodevelopmental disorders than in the control group, which agreed with other studies [19–21]. In Egypt, Shehata et al. [20] reported anti-*Toxoplasma* IgM and IgG seropositivity of 16.5% and 50%, respectively, among children with neurodevelopmental disabilities. El-Beshbishi et al. [21] reported anti-*Toxoplasma* IgM and IgG seropositivity of 20% and 38.3%, respectively, among children with neurodevelopmental disorders. Besides, anti-*Toxoplasma* IgG was detected in 42% of mentally retarded children [22]. A study conducted to investigate *T. gondii* prevalence among pregnant women in Egypt recorded a rate of 22.9% [23].

In acute infection with *T. gondii*, the specific IgM antibodies are released and initially detected. In most cases, IgM becomes negative within a few months later [24, 25]. Anti-*Toxoplasma* IgG antibodies usually appear during the first two weeks of the infection. They reach the highest level after one to two months and then decline gradually; however, they can persist for life [25].

In our study, no statistical significance was detected among various age groups for *T. gondii* seropositivity. Zhou et al. [26] reported that there is no relation between the seropositivity of the anti-*Toxoplasma* antibodies and the increase in age.

In this study, there is no significant difference in the seropositivity of the anti-*Toxoplasma* antibodies between male and female children. However, regarding the residence of the participants, the rates of anti-*Toxoplasma* seropositivity were significantly higher in patients and control children who lived in rural areas than the urban ones. This was in agreement with other studies [27, 28]. Additionally, in the patient group, a significantly high *T. gondii* antibody positivity rate was detected in association with the history of contact with soil and contact with cats. Higher prevalence of toxoplasmosis in rural areas could be linked to individuals' living patterns with more susceptibility to infection, as suitable environmental conditions for sporulation of *Toxoplasma* oocysts [28].

The clinical manifestation of congenital toxoplasmosis is widely variable from asymptomatic to severe manifestations in the newborn. Neonates could suffer from various neurological lesions or chorioretinitis. Those manifestations could appear years after birth during adolescence or even in early adulthood [29].

Diagnosis of congenital infection had relied on neonatal screening and detection of IgM, especially in absence of antenatal treatment [30]. However, careful interpretation of IgM antibody positivity should be carried out, considering the possibility of their persistence for years. Performing an IgG avidity test in addition to the detection of IgM antibodies was highly recommended to confirm the acute recent infection with *T. gondii* [31, 32]. Unfortunately, in our study, we did not perform IgG avidity to confirm acute infection, especially in the older children patients. We relied on the detection of IgM and IgG antibodies.

In our study, correlating toxoplasmosis positivity with neurodevelopmental disorders revealed significantly higher anti-*Toxoplasma* antibody and PCR positivity rates in hydrocephalus patients and significantly higher anti-*Toxoplasma* antibody positivity rates among children with epilepsy. Ottaru et al. [33] reported high seropositivity rates of anti-*Toxoplasma* IgG antibodies in infants with hydrocephalus. Higher seropositivity of anti-*Toxoplasma* IgG antibodies in children with epilepsy was recorded by other studies [34, 35]. In the study, no significant association between the seropositivity of anti-*Toxoplasma* antibodies was observed among children with attention deficit hyperactivity disorder (ADHD) or autism, which agreed with other studies [20, 36]. However, Prandota et al. [19] and Lam et al. [37] demonstrated significantly high anti-*Toxoplasma* antibodies in children with autism and ADHD, respectively. *T. gondii* parasite can directly affect neurotransmitter levels owing to its tyrosine hydroxylase encoding the gene for dopamine biosynthesis [37], constituting a probable link to ADHD and autism development.

*T. gondii* multiplication inside the brain tissues might obstruct the aqueduct of Sylvius resulting in hydrocephalus. The presence of toxoplasmosis tissue cysts in neurons and glial cells, with the formation of glial nodules, could trigger epilepsy [38].

In our study, nested PCR for amplification of the *Toxoplasma gondii* GRA6 gene showed an 11.1% positivity rate in the patient group. About 12% positivity rate of *Toxoplasma* using nested PCR of GRA6 gene was documented in another study in Egypt [39]. However, Eldeek et al. [40] recorded a higher prevalence rate of *T. gondii* (58.6%) using nested PCR of the same coding region.

Among cancer patients in China, Wang et al. [18] revealed a 3.55% positivity rate of *T. gondii* by nested PCR at the GRA6 locus. On the other hand, no positive result was obtained from amplification of the GRA6 gene in patients with ocular toxoplasmosis in Indonesia [41].

The initial dissemination of *T. gondii* tachyzoites is limited to less than 20 days from the beginning of infection, but this duration may vary according to the parasite genotype and the host immune response [32]. After the formation of tissue cysts, excystation occurs making the detection of blood parasites relatively difficult. However, incomplete eradication of the genetic component of the parasite may result in positive PCR findings in the absence of viable parasites. The negative PCR in many samples could be attributed to the presence of a low amount of parasitic DNA in peripheral blood [42]. Moreover, the low positivity rate of GRA6-based PCR might be due to the presence of this gene in a single copy in the *Toxoplasma* genome. Using multicopy genes as the B1 gene provides higher PCR sensitivity with a positive rate of up to 62% [43]. The selection of the GRA6 gene in our study was explained by its higher degree of polymorphism sufficient for the assessment of genetic diversity of *Toxoplasma* than other markers [16, 17].

In our study, MseI restriction digestion of positive PCR samples from children with neurodevelopmental disorders revealed that 80% had *T. gondii* genotype I and 20% of those children displayed RFLP atypical patterns without any significant association with specific clinical presentations. The positive PCR sample from the control group exhibited no digestion pattern, which might indicate insufficient amounts of extracted parasite DNA affected successful genotyping [44]. In agreement with our results, Tolba et al. [39] reported that out of 12 PCR-positive cases using GRA6, seven samples (58.3%) belonged to *T. gondii* genotype I, and the other five cases (41.7%) gave RFLP pattern distinct from typical ones.

Nassef et al. [45] detected *Toxoplasma* genotype I in 34.6% of PCR-positive samples from pregnant females with obstetric complications using nested PCR-RFLP at the SAG2 locus. Additionally, there was a predominance of genotype I in a study done by Eldeek et al. [40] from females with complicated pregnancies using multilocus RFLP analysis. In contrast to our findings, type II (87%) and type I (13%) were isolated from cases with abortion and intrauterine fetal death using nested PCR-RFLP for the SAG2 gene [46].

Studies demonstrated that congenital toxoplasmosis was associated with genotype I [47] and genotype II strain [48] in Tunisia.

Genotyping of *Toxoplasma* from ocular fluid samples in patients with uveitis in Indonesia by analysis of SAG2 and GRA6 loci showed that all samples were of type III allele [41].

A study in the United States revealed a high prevalence of atypical patterns (43.9%) besides type II (43.9%) and type III (12.2%) lineages using microsatellite analysis [49]. This genetic diversity was higher compared to a study from France where type II represented more than 90% of the genotypes [50].

Genotype I of *Toxoplasma* induces the production of proinflammatory cytokines that are significantly associated with severe tissue damage [51]. The inability to relate a certain *T. gondii* strain to a specific neurodevelopmental disorder emphasized that the disease outcome could be affected by other factors, such as the parasite load and genetic or immunologic background of the host [32]. Moreover, the recognition of atypical or recombinant strains could be attributed to the transmission of toxoplasmosis between wide ranges of intermediate hosts without the need for definitive hosts leading to the spread of clonal and nonclonal lineages [52]. The lack of representation of type II in our work could be explained by the dependence on a single locus for analysis [47].

## 5. Conclusion

In conclusion, our study revealed that the seropositivity rates of anti-*Toxoplasma* IgM, IgG, and combined IgM and IgG antibodies in children with neurological disorders were 10%, 23.3%, and 2.2%. Residency in rural areas represented a potential risk factor for toxoplasmosis among children. Also among the patient group, contact with soil and contact with cats were significantly associated with toxoplasmosis seropositivity. RFLP analysis of *T. gondii* GRA6 locus revealed that the most dominant genotype in the studied children's neurodevelopmental disorders was the virulent type I genotype in addition to atypical genotypes. For further characterization of *T. gondii* genotypes in Egypt, multilocus nested PCR or microsatellite analysis on a larger number of samples from different hosts is needed.

## Figures and Tables

**Table 1:** Toxoplasmosis seroprevalence and molecular positivity rates among the patient group (children with neurodevelopmental disorders) and control group.

Parameter	Study participants		*P* value
	Patient group (*n* = 180)	Control group (*n* = 180)	
Overall anti-Toxoplasma antibodies seroprevalence *n* (%)	64 (35.6)	21 (11.7)	<0.001
Anti-toxoplasma IgM seropositivity *n* (%)	18 (10)	2 (1.1)	0.04
Anti-toxoplasma IgG seropositivity *n* (%)	42 (23.3)	18 (10)	<0.001
Anti-toxoplasma IgM and IgG seropositivity *n* (%)	4 (2.2)	1 (0.5)	0.78
Anti-toxoplasma IgG concentration (IU/ml)∗	309.83 (33.9–409.65)	61.13 (35.76–97.8)	<0.001
Positive PCR for Toxoplasma DNA *n* (%)	20 (11.1)	1 (0.5)	0.03

*n*: number of participants. ^*∗*^Data was represented as medium and range (Min–Max).

**Table 2:** Seropositivity of *T. gondii* infection in relation to risk factors among the patient (children with neurodevelopmental disorders) and control groups.

Variable	Patient group (*n* = 180)				Control group (*n* = 180)			
	Tested *n*	Positive *n*	%	OR^*∗*^ (95% CI)	Tested *n*	Positive *n*	%	OR (95% CI)
Age group								
Neonate	37	18	48.6	1.41 (0.53–3.71)	31	4	12.9	0.64 (0.17–2.47)
Infant	42	9	21.4	0.23 (0.03–1.85)	50	7	14	0.47 (0.06–3.87)
Child	101	37	36.7	0.79 (0.09–6.5)	99	10	10.1	0.35 (0.04–2.88)

Sex								
Male	76	20	26.3	0.6 (0.17–2.47)	82	9	11	0.97 (0.91–1.03)
Female	104	44	42.3		98	12	12.2	

Residence								
Urban	48	8	16.7	4.3 (2.12–9.7)	51	2	3.9	2.15 (1.06–4.39)
Rural	132	56	42.4^b^		129	19	14.7^b^	

Bottle feeding								
Yes	38	9	23.7	0.63 (0.18–2.27)	25	2	8	0.41 (0.04–4.63)
No	142	55	38.7		155	19	12.2	

Drink raw milk								
Yes	30	5	16.7	0.20 (0.03–1.57)	37	5	13.5	0.71 (0.15–3.26)
No	150	59	39.3		143	16	11.2	

Eating undercooked meat								
Yes	34	7	20.6	0.59 (0.07–4.81)	46	5	10.9	0.12 (0.02–0.92)
No	146	57	39		134	16	11.9	

Contact with soil								
Yes	63	36	57.1^b^	6.59 (3.2–13.7)	46	9	19.6	1.28 (0.31–5.33)
No	117	28	23.9		134	12	8.9	

Contacts with cats								
Yes	78	34	43.6^a^	1.48 (0.57–3.8)	79	11	13.9	1.11 (0.383–3.24)
No	102	30	29.4		101	10	9.9	

*n*: number of participants, ^*∗*^ OR (95% CI) : Odd Ratio (95% confidence interval), a: statistical significant at *P* < 0.05, *b:* statistical significant at *P* < 0.001, neonate <28 days old, infant: 28 days to 1 year old, child: 1–11 years old.

**Table 3:** Seropositivity of *T. gondii* infection in relation to the neurodevelopmental disorders in the patient group.

Neurodevelopmental disorders	Patient group (*n* = 180)		
	Tested *n*	Positive *n*	%
Hydrocephalus	26	18^a^	69.2
Microcephaly	23	2	8.7
Cerebral palsy	44	6	13.6
Epilepsy	51	31^b^	60.8
Mental retardation	16	2	12.5
ADHD	14	4	28.6
Autism	6	1	16.7

*n*: number of participants. ^a^Statistical significant at *P* < 0.05. ^b^Statistical significant at *P* < 0.001, ADHD: attention deficit hyperactive disorder.

**Table 4:** *Toxoplasma gondii* GRA6-RFLP genotypes in relation to neurodevelopmental disorders.

Positive PCR cases (*n* = 20)	*Toxoplasma gondii* genotypes	
	Genotype I (*n* = 16)	Atypical pattern (*n* = 4)
Hydrocephalus (*n* = 7)	5	2
Microcephaly (*n* = 2)	2	0
Cerebral palsy (*n* = 3)	2	1
Epilepsy (*n* = 5)	5	0
Mental retardation (*n* = 1)	1	0
ADHD (*n* = 2)	1	1

*n*: number of patients, ADHD: attention deficit hyperactive disorder.

## Data Availability

The data used to support the findings of the study can be obtained from the corresponding author upon reasonable request.
